# Maximum Isokinetic Eccentric Elbow Flexor Muscle Force Can Be Estimated Using Maximum Isometric Contraction Force

**DOI:** 10.7759/cureus.70878

**Published:** 2024-10-05

**Authors:** Riku Yoshida, Kazuki Kasahara, Yuta Murakami, Masatoshi Nakamura

**Affiliations:** 1 Rehabilitation Medicine, Maniwa Orthopedics Clinic, Niigata, JPN; 2 Human Movement and Medical Sciences, Niigata University of Health and Welfare, Niigata, JPN; 3 Physical Therapy, Nishikyushu University, Saga, JPN

**Keywords:** eccentric contraction, elbow flexor, lengthening contraction, maximal voluntary contraction, muscle thickness

## Abstract

Purpose

It is difficult to measure maximal isokinetic eccentric (ECC) muscle strength in the sports field. This study aimed to investigate whether elbow isometric (ISO) flexion muscle strength or muscle thickness (MT) can be used to estimate elbow ECC flexion muscle strength.

Material and methods

Maximal muscle strength and muscle thickness (MT) were measured in the elbow flexor muscle group of 147 healthy adults (age: 21.3±0.8 years, height: 167.3±8.6 cm, body mass: 61.4±10.6 kg: 99 males and 48 females). Both isometric contraction (ISO) and eccentric contraction (ECC) of elbow flexion muscle strength were measured using an isokinetic dynamometer. The ultrasound measured MT at 50% of the distance from the upper arm to the lateral epicondyle. We performed the multiple regression analysis with elbow ECC flexion muscle strength as the dependent variable and gender, age, height, body mass, elbow ISO flexion muscle strength, and MT as the independent variables.

Results

Multiple regression analysis revealed a coefficient of determination R^2^ value of 0.89 and an adjusted R^2^ value of 0.89 (p<0.01). In addition, the independent variables elbow ISO flexor strength (p<0.01, standardized coefficient β=0.94; p<0.01, standardized coefficient β=0.89) and muscle thickness (p<0.05, standardized coefficient β=0.07) were identified as significantly associated factors.

Conclusions

The results suggest that it is possible to estimate elbow ECC flexion muscle strength using only elbow ISO flexion muscle strength and that ECC flexion muscle strength can be estimated more accurately by adding muscle thickness of the elbow flexor muscle group.

## Introduction

There are many studies on the amount of load and frequency of intervention in resistance training (RT). The American College of Sports Medicine (ACSM) guidelines recommend 8-12 repetitions at 60-80% of one repetition maximum (1RM) [1]. RT is usually performed concerning this guideline, and intensity and frequency are considered important to achieve the effects of muscle strength increase and muscle hypertrophy. Schoenfeld et al. [2] reported that in RT, the setting of training intensity is a major factor influencing the effect of RT in terms of increases in muscle strength and skeletal muscle mass. However, since the 1RM measurement uses concentric (shortening) contraction (CON) muscle strength as a guide for setting the load, the training intensity may be insufficient during the eccentric (lengthening) contraction (ECC) phase of RT.

In ECC-only resistance training (ECC-RT), it is necessary to measure ECC maximal muscle strength to establish optimal muscle strength. However, isokinetic muscle strength is difficult to measure in medical and sports settings because it requires special equipment such as a dynamometer. In rehabilitation and sports fields, muscle strength can be easily measured using isometric contraction (ISO) without needing a dynamometer or specialized machines (e.g., manual muscle test). Therefore, it is necessary to establish a method for estimating ECC muscle strength that can be easily evaluated. To the best of our knowledge, no previous studies demonstrated a relationship between maximal ISO muscle strength and maximal ECC muscle strength. Knapik et al. [3] investigated the correlation of muscle strength of knee extensors, knee flexors, elbow extensors, and elbow flexors from 352 male volunteers tested with ISO and isokinetic muscle strength using a modified Cybex II instrument. Similarly, Lucena et al. [4] investigated the relationship between ISO strength and CON strength of the shoulder rotator cuff. As a result, the correlation between ISO peak torque and CON peak torque was from r=0.49 to 0.95. Therefore, measuring ISO muscle strength may allow estimation of ECC muscle strength, which is also isokinetic, because of the observed relationship between ISO muscle strength and CON muscle strength.

Maughan et al. [5] showed a significant positive correlation between muscle strength and cross-sectional area (CSA) of knee extensors in both male and female groups. As mentioned in previous studies, ECC muscle strength has been reported to be greater than ISO muscle strength [6,7]. The previous study investigated muscle thickness and ISO muscle strength. They reported a significant positive correlation between muscle thickness (MT) of the vastus medialis and rectus femoris muscles and knee joint ISO extension muscle strength at a knee angle of 90 degrees [8]. Additionally, Akagi et al. [9] investigated that the relationship between ISO muscle strength in elbow flexion and MT x upper arm circumference in 26 men and eight women. The results reported a significant correlation for the two variables (p<0.001). Therefore, the ECC may be estimated more accurately by MT measurement or a combination of ISO muscle strength and MT measurements. Abe et al. [10], in a brief review, suggested that anterior thigh MT measured by ultrasound is a variable predictor for assessing maximum knee extension muscle strength, similar to the results of muscle CSA measured by MRI/CT.

This study aimed to investigate whether elbow ISO flexion muscle strength and MT could be used to estimate elbow ECC flexion muscle strength. We hypothesized that it would be possible to estimate elbow ECC flexion muscle strength from elbow ISO flexion muscle strength.

## Materials and methods

Participants

Maximum muscle strength was assessed in 147 healthy adults (age: 21.3±0.8 years, height: 167.3±8.6 cm, body mass: 61.4±10.6 kg), including 99 males and 48 females, in the dominant elbow flexor muscle group. This study excluded participants with upper extremity orthopedic problems, a history of recent surgery, or resistance training during the previous 6 months.

All participants were informed of the content of the study, and their consent was obtained in advance. The study was conducted with the consent of the Ethics Committee of Niigata University of Health and Welfare.

Muscle strength measurement

Muscle strength was measured using a versatile muscle function evaluation and training device (Biodex system 3.0, Biodex), and elbow ISO flexion muscle strength was performed according to previous studies measuring elbow ISO and ECC flexion muscle strength [11,12]. One week prior to the measurements, all participants took part in a session to become acquainted with the maximal voluntary contraction (MVC)-ISO, MVC-CON, and MVC-ECC torque measurements. The elbow joint flexion angle was performed at a 90° angle. After one 60% practice of the task movement before the measurement, two three-second contractions were measured. The participants were encouraged to exercise their maximum muscle strength during the measurement. After the first measurement, a one-minute break was taken, and the second measurement was performed. The maximum value of two measurements was used for statistical analysis. The elbow joint ECC flexion muscle strength was determined by setting the total elbow joint flexion range of motion from 10° to 100° and the angular velocity at 30°/sec. Before the measurement, the elbow joint ECC flexion muscle strength movements were practiced once at about 60% each and then measured three times consecutively. The examiner encouraged the participant to exercise maximum muscle strength during the measurement. The maximum value of the three measurements was used for statistical analysis.

Muscle thickness (MT) measurement

Based on previous studies [11,12], muscle thickness measurements were performed on the biceps brachii and brachioradialis muscles in the dominant elbow flexor muscle groups. B-mode ultrasonography was used with an 8 MHz linear probe (LOGIQ e V2; GE Healthcare Japan). The ultrasound intensity was 78.0, the frequency was 8.0 MHz, and the depth was 6.0 cm. In the measurements, the pressure of the probe against the skin was kept as low as possible, and the same examiner performed all measurements. The measurement site was 50% from the acromion of the lateral epicondyle of the humerus. The participants lay supine in bed, with the dominant arm relaxed and the forearm in an external rotation position. Ultrasound measurements were repeated twice, and muscle thickness of the biceps and brachialis muscles was measured as the distance from the inner edge of the fascia to the humerus.

Statistical analysis

The statistical analysis was performed using SPSS 28.0J (SPSS Japan, Inc.). The Kolmogorov-Smilov test was used to test normality. Differences in elbow ISO flexion and ECC flexion muscle strength were compared using a paired t-test. In addition, considering the influence of gender, age, height, and body mass on the relationship between elbow ECC muscle strength and each measured item, we performed a partial correlation analysis using the participant's gender, age, height, and body mass as control variables. Multiple regression analysis was then conducted using elbow ECC flexion muscle strength as the dependent variable and elbow ISO flexion muscle strength and muscle thickness, which showed a significant correlation with elbow ECC flexion muscle strength as independent variables. To prevent multilinearity among independent variables in multiple regression analysis, the variance inflation factor (VIF) of each independent variable was checked to see if the VIF was less than five. The significance level was set at 0.05.

## Results

The measured elbow ISO flexion muscle strength (45.9 ± 13.5 Nm) and ECC flexion muscle strength (50.6 ± 13.4 Nm) are shown as mean ± standard deviation (Table 1). The results of this study showed that elbow ECC flexion muscle strength was significantly greater than elbow ISO flexion muscle strength by 11.7 ± 11.7% (p<0.01).

**Table 1:** Different contraction types of elbow flexor muscle torque * significant difference compared to ISO ISO - elbow joint isometric flexor strength; ECC - elbow joint eccentric flexor strength

Contraction type	Torque (Nm)
ISO	45.9 ± 13.5
ECC	50.6 ± 13.4*

Table 2 shows the results of the multiple regression analysis with the dependent and independent variables as in the partial correlation analysis. In model 1, the probability of significance was p < 0.01, the coefficient of determination R2 value was 0.89, and the adjusted R2 value was 0.89. In model 2, the coefficient of determination R2 value was 0.89, and the adjusted R2 value was 0.89. In addition, elbow ISO flexion muscle strength (p<0.01, standardized coefficient β=0.94), an independent variable in model 1, was extracted as a significantly associated factor. The formula was: elbow ECC flexion muscle strength = elbow ISO flexion muscle strength × 0.938 + 7.47. On the other hand, in model 2, the independent variables of elbow ISO flexion muscle strength (p<0.01, standardized coefficient β=0.89) and muscle thickness (p<0.05, standardized coefficient β=0.07) were significantly related factors. The formula was: elbow ECC flexion muscle strength = elbow ISO flexion muscle strength × 0.885 + muscle thickness × 0.251 + 3.973.

**Table 2:** Factors associated with elbow eccentric (ECC) flexion muscle torque MT - elbow flexor muscle thickness; VIF - variance inflation factor

	Unstandardized coefficient		Standardization coefficient	Significant probability	95% confidence interval		Statistics of collinearity	
	B	Standard error	β	p-value	Lower limit	Upper limit	Tolerance level	VIF
Model 1								
Constant	7.470	1.305	-	< .001>	4.890	10.04	-	-
Isometric contraction torque (Nm)	0.938	0.027	0.944	< .001>	0.885	0.992	1	1
Model 2								
Constant	3.973	2.369	-	0.07	-0.326	8.273	-	-
Isometric contraction torque (Nm）	0.885	0.04	0.891	< .001>	0.810	0.960	0.507	1.972
MT (mm)	0.251	0.136	0.076	< .005>	0.003	0.499	0.507	1.972

## Discussion

This study aimed to investigate whether ECC muscle strength could be estimated by easily measured elbow flexion ISO muscle strength. The results suggested that ISO alone could be used to estimate ECC muscle strength (adjusted R2 value was 0.89) and that adding MT (adjusted R2 value was 0.89) could be used to estimate it more accurately. The results support the hypothesis. Although previous studies have reported that ECC muscle strength is greater than ISO muscle strength [6,7], this is the first study to examine the estimation of ECC muscle strength by ISO muscle strength and MT using multiple regression analysis.

In a previous study [6], ECC muscle strength was reported to be 13.5% greater than ISO muscle strength in elbow flexors. Similarly, other previous studies have reported that ECC muscle strength is approximately 15% greater than ISO muscle strength in the elbow flexor muscle group [7]. Similar to previous studies [6,7], this study also shows that ECC muscle strength is more than 10% greater than ISO.

In model 1, the coefficient of determination R2 value was 0.89, and the adjusted R2 value was 0.89. In model 2, the coefficient of determination R2 value was 0.89, and the adjusted R2 value was 0.89 (Table 1). The results suggest that ECC muscle strength is also a factor that can be estimated in ISO muscle strength. The study did not measure the detailed central and peripheral mechanisms in the force development of the muscles for each contraction mode. Therefore, we cannot discuss the details, but we believe that contraction mode affects muscle force. Although ISO, CON, and ECC are the main contraction modes of muscle, previous studies reported that they are similar concerning the mechanism of each contraction mode. In the muscle contraction modes, these studies reported similar muscle exertion mechanisms in the E-C coupling and in the sliding theory [13-16]. Therefore, it was suggested in this study that elbow ECC flexion muscle strength could be estimated from the elbow ISO flexion muscle strength.

The previous study investigated muscle thickness and ISO muscle strength of knee extensors. They reported a significant positive correlation between men (r=0.41, p<0.01) and women (r=0.22, p<0.01) in the MT of the vastus medialis and rectus femoris muscles and knee joint ISO extension muscle strength [8]. Computed tomography (CT) and magnetic resonance imaging (MRI) are the most common methods for measuring muscle thickness and cross-sectional area. Still, MT can also be easily measured with the ultrasound used in this study. Considering the time-consuming and costly evaluation of muscle thickness using CT and MRI in sports and rehabilitation settings, we believe that measurement using ultrasound is important. This study suggested that in addition to measuring elbow joint ISO flexion muscle strength, elbow joint ECC flexion muscle strength could be estimated more accurately by measuring muscle thickness of the elbow joint flexor muscle group imaged with an ultrasound imaging system.

ECC-only RT requires approximately 50% less oxygen uptake than concentric CON-only RT [17]. Also, previous studies have reported that ECC-only RT is more effective than CON or ISO-only RT in increasing muscle strength and hypertrophy [18-20]. In addition, the importance of ECC training in preventing hamstring tears and anterior cruciate ligament injuries, as well as in rehabilitation programs, has been highlighted in clinical settings [21]. Moreover, Yoshida et al. [22] showed that six repetitions of submaximal ECC contraction (2/3 maximal ECC strength) using a dumbbell five days per week for four weeks could increase muscle strength and thickness. However, the ECC strength measurement requires special equipment such as a dynamometer, and a big issue is difficult to measure in sports and clinical settings. As mentioned above, measuring eccentric muscle strength is important for setting training loads and constructing treatment strategies. This study suggests that elbow joint ECC flexion muscle strength can be estimated by elbow joint ISO flexion muscle strength alone and that ECC flexion muscle strength can be estimated more accurately by adding muscle thickness of the elbow joint flexor muscle group. It is important to put a firm load on the ECC phase in the resistance training. Therefore, measuring maximum ISO muscle strength and MT, rather than performing maximum ECC muscle strength, which is difficult to measure, could provide useful information for measuring ECC strength and setting training loads in sports and clinical settings.

This study has several limitations. First, only the elbow flexor muscle group was targeted. Future studies should examine whether the relationship between ISO and ECC muscle strength in the lower limbs and trunk muscles is similar to the results of this study. Second, the measurement equipment. The device used to measure elbow flexion muscle strength in this study was a multi-purpose muscle function evaluation device, which is not widely used. In the future, it is necessary to examine whether the same results can be obtained with dumbbells and hand-held dynamometers as in the present study. Third, the male/female ratio. The male-to-female ratio of the participants in this study was 99 males and 48 females, with more males than females. Therefore, it is necessary to clarify the relationship between ISO muscle strength, ECC muscle strength, and MT when the ratios of men and women are equal. Fourth, only healthy university students were included in the study. This study did not measure ECC muscle strength in older adults. Since RT is performed by both men and women of all ages, it is necessary to examine whether ECC muscle strength can be estimated in older adults as well as in the present study. Fifth, the elbow ISO muscle strength was measured only at 90°, indicating that the muscle strength exerted by the ECC muscle differs depending on the joint angle [23]. Therefore, it is possible that the 90° angle in this study was not sufficient. In the future, it is necessary to consider measuring ISO muscle strength by considering at which angle maximum muscle strength can be exerted in the exerted muscle strength for each joint angle in ECC muscle strength.

## Conclusions

The study found that elbow ECC flexion muscle strength (50.6 ± 13.4 Nm) was significantly greater than elbow ISO flexion muscle strength (45.9 ± 13.5 Nm) by 11.7 ± 11.7% (p<0.01). Multiple regression analysis results showed that in model 1, elbow ISO flexion muscle strength was a significant predictor of ECC flexion muscle strength, with the formula: ECC flexion = ISO flexion × 0.938 + 7.47. In model 2, both elbow ISO flexion muscle strength and muscle thickness were significant predictors, with the formula: ECC flexion = ISO flexion × 0.885 + muscle thickness × 0.251 + 3.973. Both models had high R² values (0.89).
